# Evolutionary innovation using EDGE, a system for localized elevated mutagenesis

**DOI:** 10.1371/journal.pone.0232330

**Published:** 2020-04-30

**Authors:** Xiao Yi, Romas Kazlauskas, Michael Travisano

**Affiliations:** 1 BioTechnology Institute, University of Minnesota, St. Paul, Minnesota, United States of America; 2 Biochemistry, Molecular Biology and Biophysics, University of Minnesota, St. Paul, Minnesota, United States of America; 3 Ecology, Evolution and Behavior, University of Minnesota, St. Paul, Minnesota, United States of America; Chang Gung University, TAIWAN

## Abstract

Mutations arising across the whole genome can hinder the emergence of evolutionary innovation required for adaptation because many mutations are deleterious. This trade-off is overcome by elevated mutagenesis to localized loci. Examples include phase variation and diversity-generating retroelements. However, these mechanisms are rare in nature; and all have narrow mutational spectra limiting evolutionary innovation. Here, we engineer a platform of **E**xperimental **D**esigned **G**enic **E**volution (EDGE) to study the potential for evolutionary novelty at a single locus. Experimental evolution with EDGE shows that bacterial resistance to a novel antibiotic readily evolves, provided that elevated mutagenesis is focused on a relevant gene. A model is proposed to account for the cost and benefit of such single loci to adaptation in a changing environment and explains their high mutation rates, limited innovation, and the rarity of localized mutagenesis in nature. Overall, our results suggest that localized mutation systems can facilitate continuing adaptive evolution without necessarily restricting the spectrum of mutations. EDGE has utility in dissecting the complex process of adaptation with its localized, efficient evolution.

## Introduction

Higher genome-wide mutation rates can both promote and impede adaptation. Adaptation requires genetic variation, and mutations are the ultimate source of all genetic variation. Newly arisen beneficial mutations contribute to adaptation by increasing in frequency due to natural selection. However, increases in mutation rate can also impede adaptation [1]. High genome-wide mutation rates increase the occurrence of both beneficial and deleterious mutations. An excess of deleterious mutations slows adaptation and potentially causes extinction [2, 3]. The effects of most mutations range from selectively neutral to deleterious [4], but even modest numbers of deleterious mutations can reduce individual viability [5] and increase the mutation load [6, 7]. Low genome-wide mutation rates minimize the number of deleterious mutations but also simultaneously reduce the number of beneficial mutations [8], slowing adaptation. Because of these complexities, a high genome-wide mutation rate can provide a short-term fitness benefit by increasing the number of beneficial mutations but incur a longer-term cost by loading the genome with deleterious mutations [9].

Localized mutators, in contrast, could circumvent some of the trade-offs associated with genome-wide mutation rates. To avoid the fitness costs of global increases in mutation rate, Fitch [10] suggested that “If the organism needs to change only a few of its genes, one would prefer to increase the mutation rate in those genes specifically.” Localizing enhanced mutation rates to specific regions reduces the burden of new deleterious mutations across the genome, while still facilitating the appearance of beneficial mutations at relevant loci. Since Fitch’s original conjecture, multiple naturally occurring mechanisms have been shown to localize mutations to specific loci, including microsatellites, recombinational switches, and transposon-mediated alterations. In microorganisms, these mechanisms are frequently associated with virulence and can provide a mechanism for immunogenic escape [11–14]. These naturally evolved mechanisms of site-specific mutation not only restrict mutations to a region but also restrict the kinds of mutations. For example, recombinational switches have only two allelic states (“on” and “off”), and microsatellite mutations primarily involve insertions/deletions of DNA repeats. The limited number of states facilitates evolutionary reversals, which is beneficial is escaping immune responses [15]. Thus, these naturally occurring mechanisms reduce the number of deleterious alleles in two ways, by confining mutations to a genomic region and by limiting the spectrum of possible mutations.

But, most naturally occurring mechanisms limit the possibility of evolutionary innovation. By restricting the scope of possible mutations, most localized mutators also prevent entirely novel mutations from occurring. We are studying the potential for evolutionary novelty at a single locus. We developed an experimental approach, **E**xperimental **D**irected **G**enetic **E**volution (EDGE), and our focus in this study is the evolution of resistance to antibiotics. Bacterial antibiotic resistance is a severe and growing problem, requiring an understanding of the evolutionary mechanisms of resistance. Previous studies have used artificial constructs to examine the scope of *in vivo* adaptation by targeted evolution [16–18], most having elevated genome-wide mutational effects even if the target of interest is a single locus. We take a different approach, EDGE (**E**xperimental **D**esigned **G**enic **E**volution), by elevating mutation rates at a single locus similar to that observed in naturally occurring localized mutation systems. Unlike other systems, natural or artificial, EDGE facilitates both evolutionary reversibility and innovation. EDGE leverages an existing mechanism of introducing mutations, error-prone DNA repair, in a simple construct. By building upon error-prone DNA repair, EDGE does not limit the spectrum of possible mutations, unlike those of many naturally occurring localized mutation systems. We develop a model showing high expected fitness costs of EDGE-like systems, which provides an explanation for their rarity in nature as well as their localized high mutation rates. In this paper, we show that localized mutation systems can facilitate continuing adaptive evolution without necessarily restricting the spectrum of mutations.

## Results

### Design of the genetic circuits

**Fig 1:**
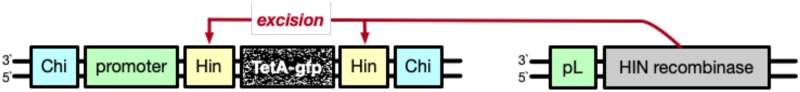
The EDGE genetic circuit to increase the mutation rate of a target gene, here a TetA-gfp fusion. A Hin specific endonuclease cleaves both DNA strands at the Hin sites (yellow). Next, resection removes the 5' strands on both sides up to the chi sites (blue). Error-prone DNA repair generates mutations in the target gene and flanking regions.

Stress-induced mutagenesis (SIM) is the basis for the EDGE directed mutation genetic circuit. In bacteria under stress, DNA repair is error-prone and increases the mutation rate [19]. In *Escherichia coli* specifically, stress-induced mutagenesis occurs following DNA double-strand breaks and repair by an error-prone DNA polymerase [20–22]. While stress-induced mutagenesis involves the entire chromosome, we constructed a genetic circuit that uses a similar approach as stress-induced mutagenesis but targets only a single gene. In our test case, this gene encodes TetA-gfp, a fusion of an efflux pump that provides resistance to tetracycline and green fluorescent protein as a reporter. The genetic circuit is on two plasmids, a target plasmid and an accessory plasmid (Fig 1). The target genetic circuit is on a low copy number plasmid pSC101, about twenty copies per cell, and an ampicillin resistance marker. The target gene is under the control of a constitutive promoter and between two short DNA sequences (Hin sites, yellow) where the double-strand breaks occur. Two chi sites (blue), which promote double-strand break repair, bookend the entire region.

The accessory plasmid encodes the M109E variant of Hin recombinase. The typical Hin recombinase from the bacterium *Salmonella enterica* catalyzes the excision or inversion of the DNA fragment intervening the two Hin sites by first creating double-strand breaks and second, repairing these breaks. In contrast, the M109E variant of Hin recombinase creates double-stranded breaks at the Hin sites, but cannot repair them [23] and thus is a Hin-specific endonuclease. The addition of isopropyl β-D-1-thiogalactopyranoside (IPTG) turns on the tac promoter that controls the expression of M109E Hin recombinase.

Targeted mutagenesis is initiated by the addition of IPTG, inducing expression of M109E Hin recombinase and thereby creating double-strand breaks at the Hin sites. The pair of chi sites flanking the operon defines the range of mutagenesis and protects the plasmid from complete degradation [23]. Upon the generation of a double-strand break, resection degrades the 5’ single strand on each side of the break stopping at a chi site (Fig 1). This process leaves an overhang of 3’ single strand on each side of the break. Then DNA polymerization fills the gap, restoring the double-strand, and repairing the break [21]. The template for repair is another, unbroken, copy of the multicopy plasmid. The polymerase for repair, DNA polymerase IV, is error-prone, so this repair introduces mutations into the newly synthesized DNA When induced, this system repeatedly introduces double-strand breaks on the target plasmid; at the same time, SIM continuously repairs these breaks. In consequence, mutations are expected to accumulate in the target region.

### Targeted increased mutagenesis

**Fig 2:**
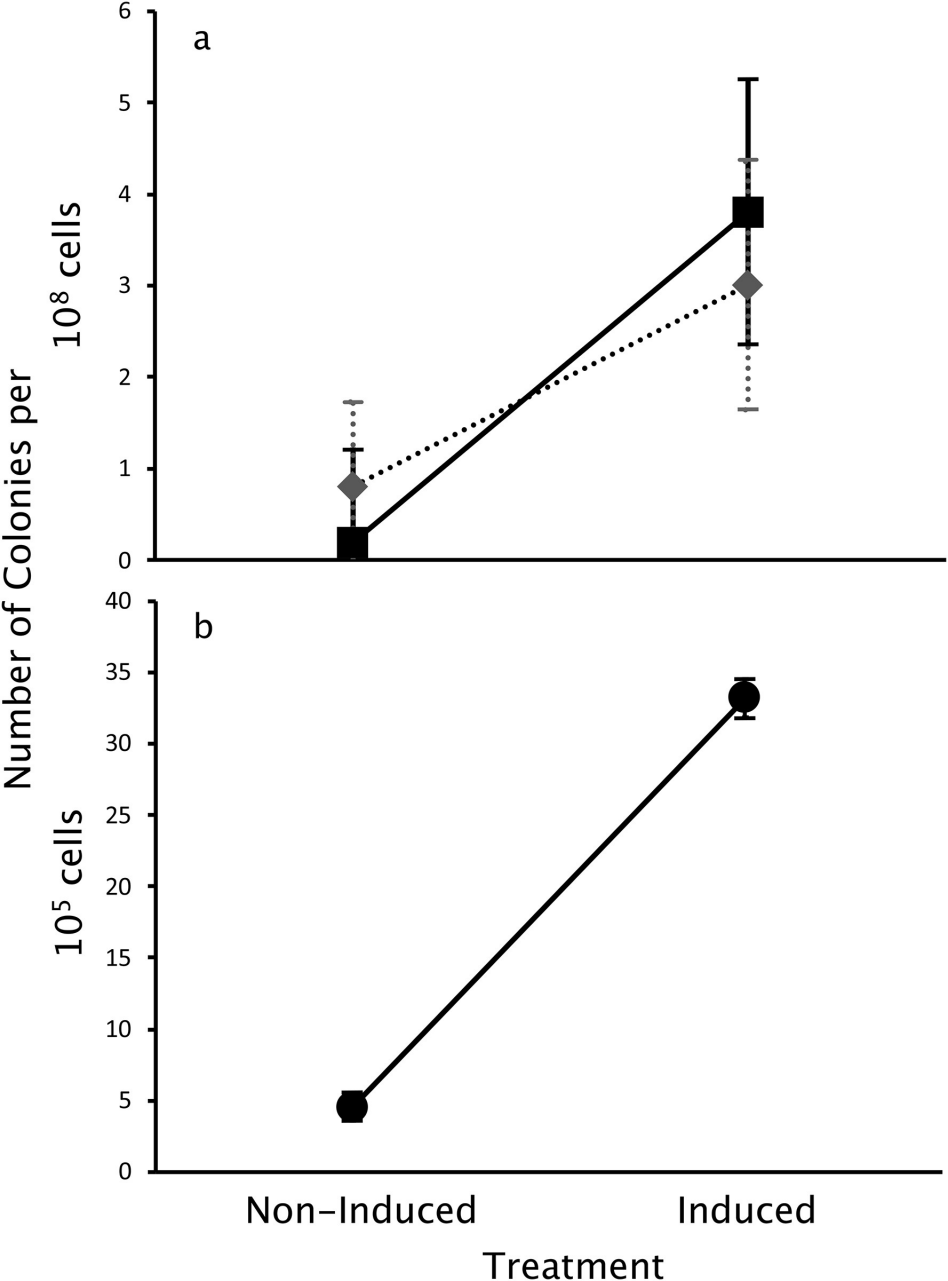
EDGE induction increases mutagenesis, especially in the *TetA*-*gfp* target gene. **a**. Growth on tetracycline plates selects for the gain of tetA efflux pump activity. EDGE induction increases the appearance of gain-of-function tetracycline resistant mutants on plates supplemented by 4 μg/mL tetracycline (squares, solid black line, p = 0.005). Growth on streptomycin also selects for streptomycin resistance, but EDGE induction only marginally increases the appearance of streptomycin resistance mutants (diamonds, dashed line, p = 0.074). The strep resistance gene is not the target locus. **b**. Growth on apramycin plates selects for the loss of tetA efflux pump activity [6]. EDGE induction increases the appearance of apramycin resistant loss of function mutants on plates supplemented by 4 μg/mL apramycin (circles, black line, p = 3.17 x 10^−11^). Means and confidence intervals (CI) are determined by two-way ANOVA. CI are standard errors.

We tested for enhanced mutagenesis using two evolutionary challenges, one involving a gain of function and a second on the loss of function. The gain-of-function challenge was tetracycline resistance, and we started with an inactive variant of the TetA efflux pump as the target gene. The inactive variant provides no tetracycline resistance and has a single nucleotide difference, G202A, from the active tetA gene. Previous research shows that tetracycline resistance gain of function requires an A202G transition, replacing the asparagine in the inactive variant with an aspartate [24]. Following IPTG induction of the EDGE genetic circuit, we observe many more colonies on tetracycline supplemented plates than without induction: 3.8 versus 0.2 mutants per 10^8^ cells (Fig 2a, square symbols). The increase in mutant colonies by induction of the EDGE genetic circuit indicates a greater than 10-fold increased mutagenic output at the target region (planned contrast, *t*_*24*_ = 3.06, p = 0.005).

In a separate experiment, we tested for enhanced mutagenesis by measuring a correlated loss of function of the TetA efflux pump. In this experiment, we started with the active form of the TetA efflux pump as the target gene. Expression of active tetA increases *E*. *coli* sensitivity to the antibiotic apramycin [25], and loss of tetA function allows those variants to grow on plates containing apramycin. Following IPTG induction of EDGE, we observe a seven-fold increase in the number of apramycin resistant mutants than without IPTG, (34.7 versus 5 mutants /10^5^ cells, Fig 2b). This loss of tetA function (and the resulting appearance of apramycin resistance) demonstrates that the enhanced mutation rate leads to loss of function in the absence of selective pressure for that function. As above, this result is consistent with a substantially increased mutation rate of the target region (planned contrast, *t*_*24*_ = 11.47, p = 3.17 x 10^−11^).

We excluded confounding false positives arising from plasmid loss, which also removes active tetA from the cells by counting only those colonies that also maintained resistance to ampicillin. The EDGE target plasmid construct contains an ampicillin resistance marker (not at the target locus), so colonies that retain ampicillin resistances still contain the plasmid. Nine out of ten of the colonies that grew on apramycin were still fluorescent according to flow cytometry. This result shows that most apramycin-resistant cells maintain the fusion protein and GFP function. Apramycin selects for loss of tetA function, which can be caused either by point mutations or indels within the tetA gene. Point mutations would keep GFP fluorescence, while most indels lead to frameshifts, which would inactivate both tetA and GFP. The 90% retention of GFP signal after selection suggests that point mutations are the most common mutation. The 10% loss of GFP signal was most likely caused by indels on tetA because the two proteins (TetA-gfp) were fused together. Indeed, dinB error-prone DNA polymerase of E. coli generates indels as well as substitutions [26].

One striking difference between the results of the two evolutionary challenges is an almost 10^4^-fold difference in the number of mutant colonies. There were many fewer gain of function tetracycline-resistant colonies observed in the first challenge than the loss of tetracycline (gain of apramycin) resistance in the second challenge. The differences occurred regardless of EDGE induction. Our results are consistent with ease of loss of tetA function relative to gain. Tetracycline resistance gain of function requires a specific mutation (A202G), while a loss of function can arise from many different mutations.

We tested the localization of EDGE induced mutagenesis using a third evolutionary challenge, spontaneous evolution of resistance to the antibiotic streptomycin. *E*. *coli* readily evolves resistance to streptomycin by a single nucleotide substitution on the chromosome [27]. The rate of streptomycin resistance evolution increased slightly with IPTG induction of Hin recombinase M109E, but the increase in rate is only marginally statistically supported (Fig 2a, dashed line; (planned contrast, *t*_24_ = 1.87, p = 0.074). There are statistically different effects of EDGE induction (*F*_2,24_ = 27.38, p = 6.41 x 10^−7^) across the three antibiotics: tetracycline (gain-of-function), apramycin (loss-of-function) and streptomycin (off-target). A direct comparison between streptomycin and tetracycline, shows insignificant statistical differences. Induction of EDGE for tetracycline increases mutation rate 16.3-fold (estimated by bootstrap, see Methods), while only 4.8-fold for streptomycin. But the 95%CI interval between the two, tetracycline—streptomycin, ranges from -3 to 26. While we can confidently support elevated mutation rates by EDGE induction for targeted genes, there is weak support for a modest increase in global mutation rates.

### Selection for increased mutagenesis

We hypothesized that increasing the Hin endonuclease activity would improve EDGE targeted mutagenesis. The endonuclease activity starts mutagenesis by generating the pair of DNA double-strand breaks (Fig 1). Using error-prone PCR, we generated a library of the accessory plasmids carrying mutagenized versions of the M109E variant of Hin recombinase gene. These plasmids were transformed into cells harboring the target plasmid with the inactive G202A version of tetA. We induced EDGE function and selection for recovery of tetracycline resistance by plating the library onto medium containing IPTG (0.2 mM) and tetracycline (4 μg/mL). We harvested the first four initial colonies, expecting the rapid appearance of tetracycline resistance is due to Hin recombinase variants with higher endonuclease activity. The accessory plasmid was extracted from these colonies, reintroduced into cells with EDGE genetic circuits, and a target tetA-gfp fusion. The variant plasmids generated 30 to 270% more mutant colonies than the unselected accessory plasmids: up to 129.1 vs. 34.7 mutants per 10^5^ cells (S1 Fig). Sequencing revealed single nucleotide substitutions in each of the Hin recombinase variants (S1 Table) consistent with a 20-fold improved endonuclease function, based on the Hin recombinase protein structure (S2 Fig). No signals for mixed sequences indicating plasmid heterogeneity were seen in Sanger sequencing.

### Evolution of expanded resistance to a novel antibiotic

**Fig 3:**
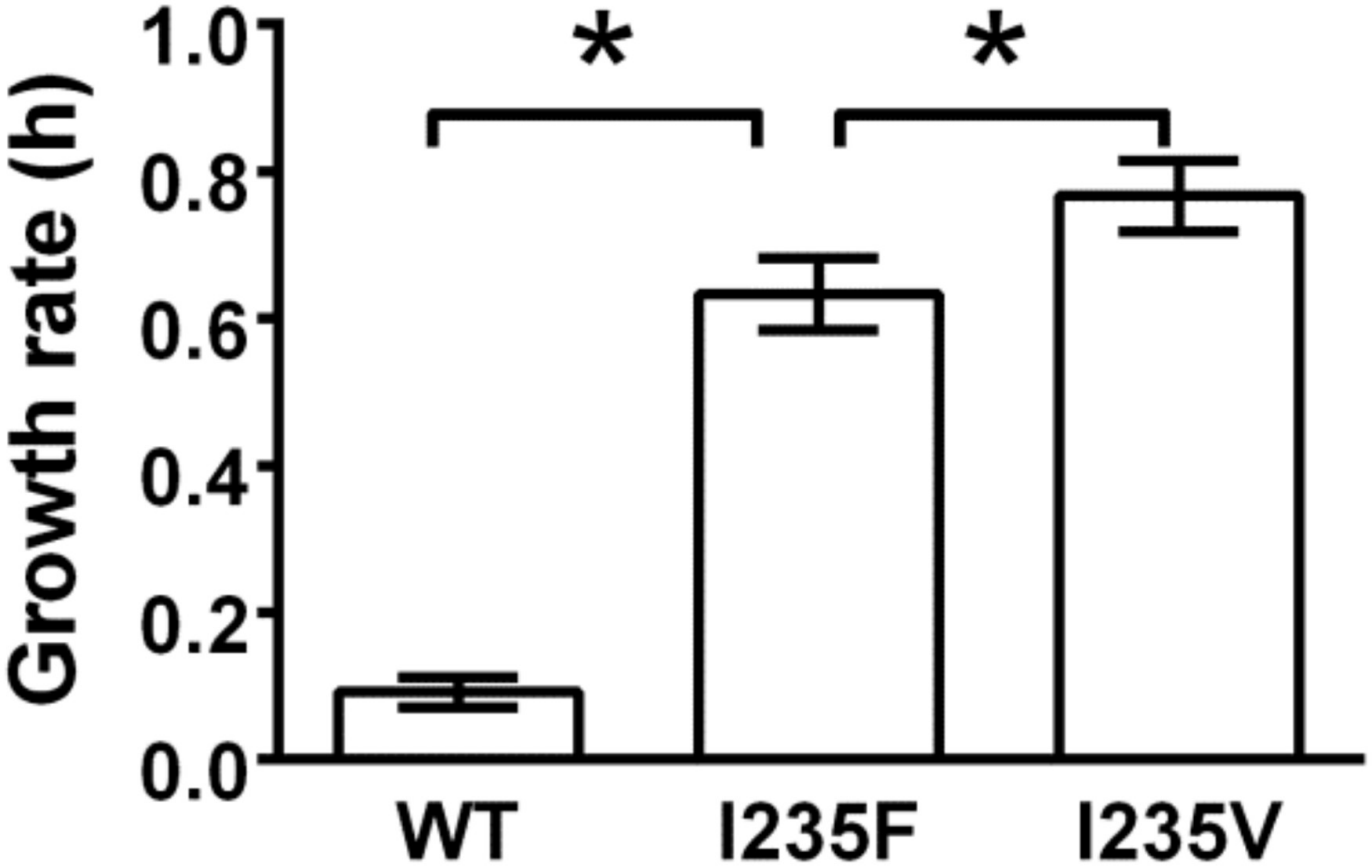
Growth rates of the ancestral genotype (WT) and two tetA antibiotic-resistant mutants. The growth rate of the EDGE derived mutant (I235V) is larger than both the “wild type” and that previously obtained by *in vitro* methods (I235F). * indicates p< 0.05.

Having validated the targeted mutation of the EDGE genetic circuit, we investigated the evolution of resistance to a novel antibiotic. Tigecycline is a tetracycline derivative and is a last-resort antibiotic because none of the existing tetracycline resistance proteins enable resistance to tigecycline [28]. Using IPTG, we induced the EDGE genetic circuit with a tetA-gfp gene as the target. After 24 hours, the cells were plated on agar containing 4 μg/mL tigecycline. Resistant colonies appeared rapidly within 16 hours of incubation at 37°C, suggesting that the resistant mutants were already present in the batch culture and did not arise from stress on the plates due to the presence of an antibiotic. Colonies that appeared to grow fastest (largest at the end of incubation period) were isolated, plasmids were extracted, and tetA region sequenced. Among the ten isolates checked, all carried mutations with three different genotypes. The mutant with isoleucine 235 replaced by valine gained the most pronounced resistance to tigecycline (minimal inhibitory concentration of 1.6 μg/mL; 0.2 μg/mL for empty cells). The unselected genotype has a growth rate of 0.1 h-1 in the presence of tigecycline, while the I235V variant is almost eight times greater, 0.79 h-1 (Fig 3).

**Fig 4:**
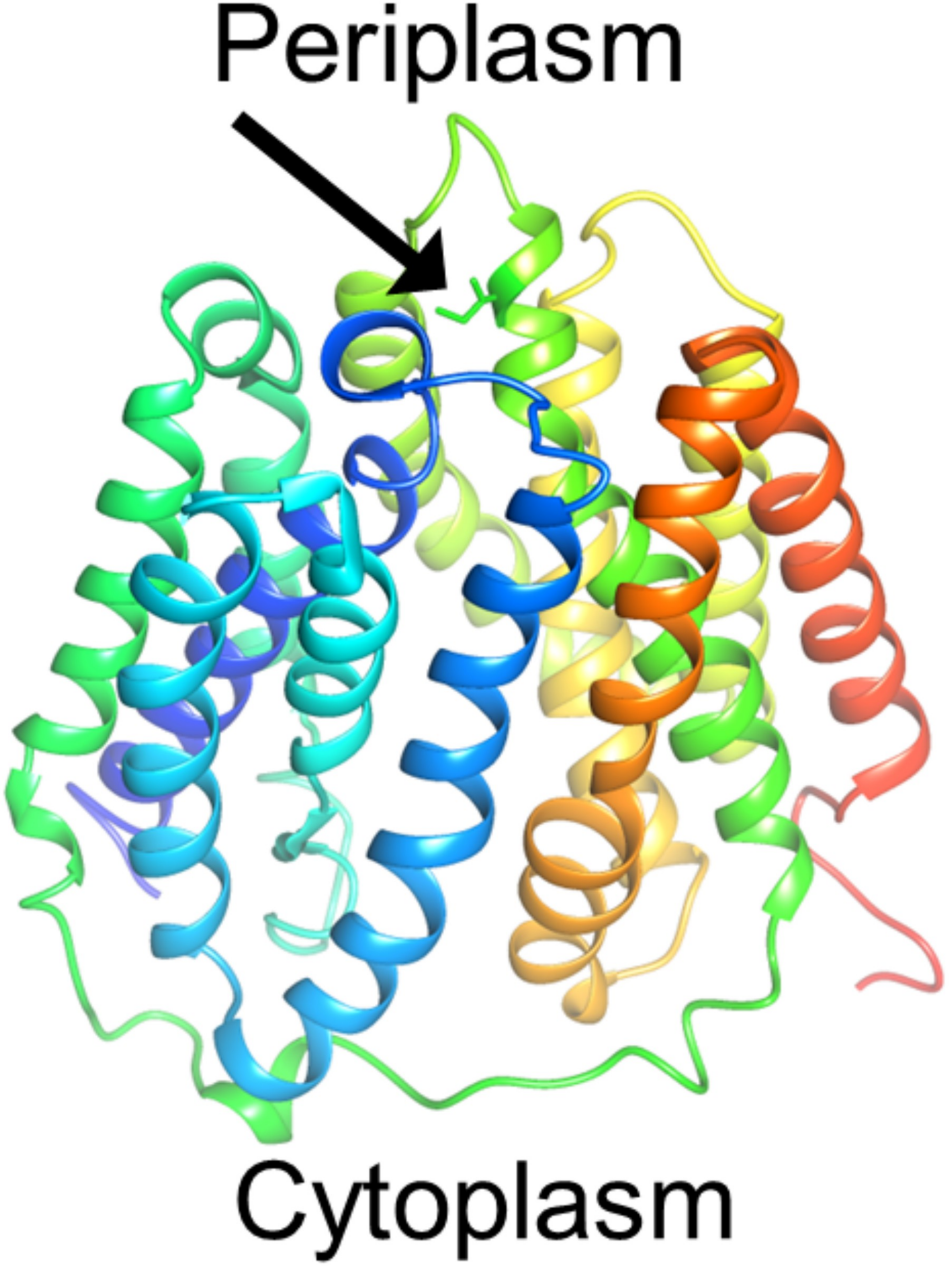
Homology model of tetA. The arrow points to the mutated residue (isoleucine 235 with side chain shown) in both traditional directed evolution and EDGE to evolve resistance to tigecycline. This mutation lies at the exit of the pump on the periplasmic side. Colors distinguish the different alpha helices for clarity. The model was created using Phyre2 with intensive mode [10].

Our observation is both similar to and different from previous research on tigecycline resistance. Previous research using PCR directed *in vitro* mutagenesis to evolve tetA for resistance to tigecycline [27], found isoleucine 235 to phenylalanine was critical for conferring the highest level of resistance. With EDGE, we also observed that the same site is critically important, but changing to valine (Fig 3). Isoleucine 235 sits at the opening on the periplasmic side of the tetA efflux pump (Fig 4). The independent discovery of substitution at this residue by the group and in this work suggests its critical role in determining substrate specificity. This coincidence also demonstrates that our system of mutagenesis has already achieved a utility of directed evolution comparable to the current standard of *in vitro* systems.

### Targeted mutagenesis has high expected fitness costs

**Fig 5:**
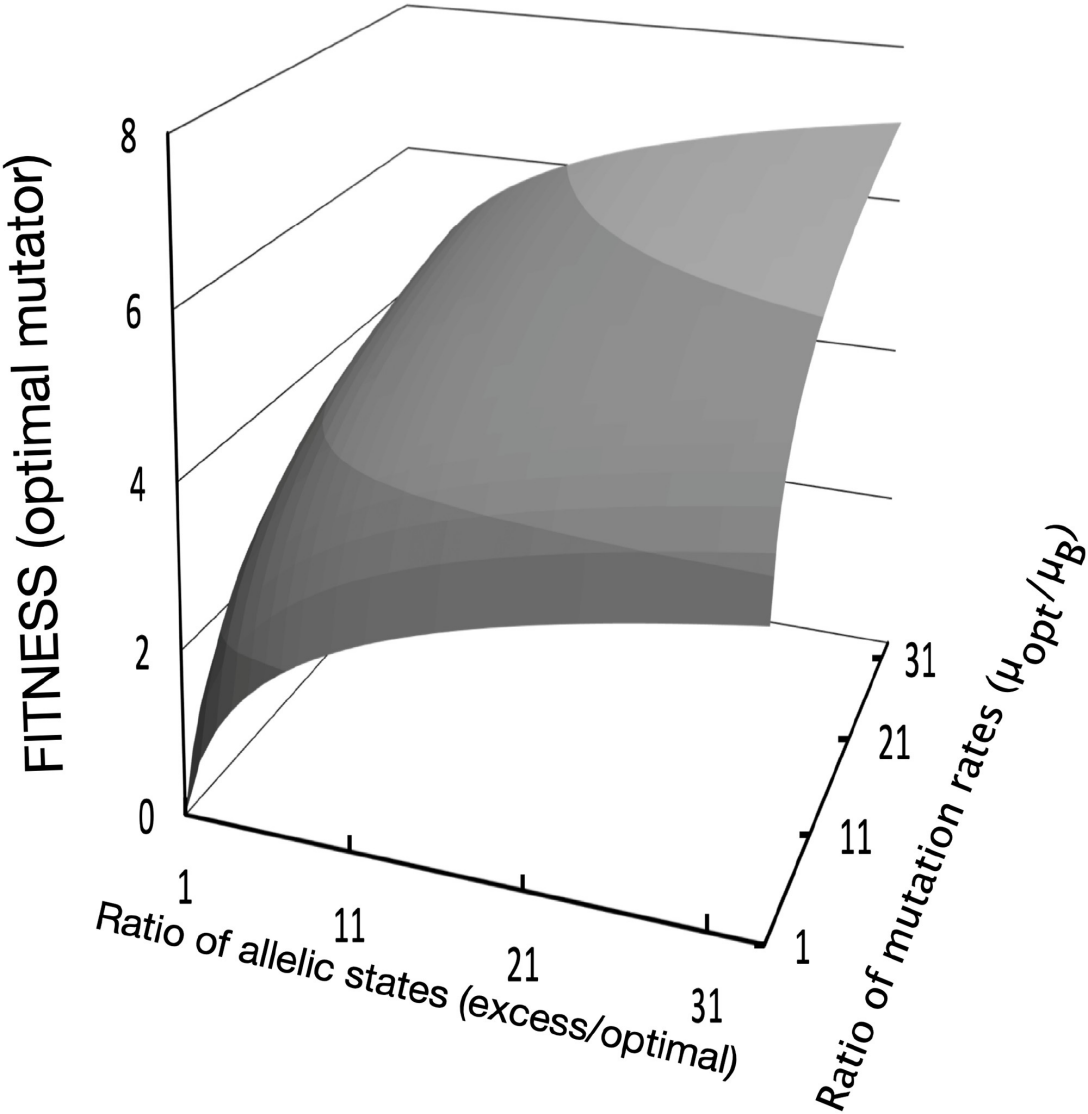
The fitness of localized mutators is strongly affected by the number of allelic states and the rate of mutagenesis to alleles. The model predicts that optimal mutators have the minimum number of necessary allelic states and high rates of localized mutagenesis, as is seen in natural systems. Fitness is determined by the difference in malthusian parameters [31, 32].

Using a simple analytical model, we show that targeted mutagenesis systems are expected to incur high fitness costs. We observe that even modest differences in the number of mutational states generate large fitness differences among mutator loci. In the model, mutations only occur during environmental shifts, and not under other conditions, thereby lowering the overall fitness costs of mutations. The optimal mutator locus in the model has a one-to-one matching of mutational states to environmental states. Only the individuals with the matching allele survive the shift to the then-new environment, and the population crashes to extinction in the absence of the matching allele. At the same time, excess mutational states are deleterious because a larger number of individuals carry maladaptive alleles. Fitness (Selection Rate Constant, r [29,30]) is higher with an optimal mutator locus (with n allelic states) relative to a locus with an excess number of states (x > n) is $r_{N/X}=ln(\frac{x}{n})$. Excess mutant allele possibilities reduce fitness, even when mutations are essential for persistence (Fig 5). This result is not strictly a consequence of the absolute mutation rates of the mutator loci, but their relative rates. Mutators with lower absolute mutation rates, but with the same number of allelic states, generate fewer mutants with the necessary beneficial allele. A locus with a higher mutation rate (A) is selectively beneficial relative to a lower mutation rate locus (B), $r_{A/B}=ln(\frac{\mu_{A}}{\mu_{B}})$, (0 < *μ*_*B*_ < *μ*_*A*_ ≤ 1), if both have the same number of allelic states. Populations are partially shielded from the deleterious effects of high mutation rates, if mutations are localized to a small genomic region and mutagenesis is strongly regulated.

## Discussion

Many complex molecular mechanisms influence local mutation rates and mutational effects. Some mechanisms mask the deleterious effects of mutations [33], some increase the proportion of beneficial mutations [34], and others increase the likelihood of rare beneficial mutations [35]. One of the challenges for understanding these mechanisms is their evolutionary basis: are they adaptations [36, 37]? In specific cases, it’s clear that selection has shaped molecular mechanisms favoring local, targeted elevated mutation rates that increase fitness. Hence, some mechanisms are adaptations. But the same molecular mechanisms that target mutation to a specific genomic region also tend to restrict the diversity of mutations, potentially limiting evolutionary innovation. Experimental Directed Genetic Evolution (EDGE) demonstrates the potential for supplement targeted evolution without necessarily constraining mutational spectra.

As expected from previous *in vitro* studies, we observe that error-prone DNA-repair mutation rates are sufficient for enhanced adaptive responses under strong selection, such as resistance to tetracycline. Two additional evolutionary challenges showed the potential for the extended evolution of the EDGE system. In one, we observed that the EDGE facilitates evolutionary reversals. The potential for easily acquired evolutionary reversal is a hallmark of most of the natural adaptive mutation systems. For example, phase variation in *E*. *coli* occurs by site-specific recombination of a DNA segment that turns “on” or “off” expression of type 1 fimbria, a major virulence factor [38, 29]. Phenotypic change by a mutational switch, rather than by regulation or gene loss, increases survival and rapid adaptation under lethal selection. Unlike transcriptional regulation, a recombinational switch leads to complete elimination of the target gene expression. This can be critical for immunogenic escape or antibiotic resistance [30]. Reacquisition of gene expression requires a phenotypically equivalent back mutation in the same locus. Such back mutations occur at high rates in adaptive (natural) mutation systems, at much higher rates than if the original change in phenotype occurred by gene loss.

We also observed the potential for extended evolution with EDGE in the evolution of a new function. The gain of resistance to tigecycline, a relatively new third-generation tetracycline derivative, is an example of evolutionary innovation. The evolution of novelty in naturally occurring adaptive mutation systems is difficult because they rely on the production of allelic variants. In most natural systems, there are only a few variants, such as the two allelic states in the phase variation system described above. While the different alleles are beneficial in different conditions, the small number of allelic variants eliminates the potential for innovation. In contrast, variants generated by a mutational hotspot are likely to be novel. But most mutations are also expected to be neutral or deleterious, which means the hotspot is less likely to be an adaptation because any evolutionary benefits are assessed over the entire mutation load acquired by all the variants.

Considering the relative simplicity of the EDGE system, why are natural loci incorporating both reversibility and innovation rare? Our analytical model shows that there are high fitness costs associated with EDGE-like systems to incorporate both reversibility and evolutionary innovation. The surprisingly narrow conditions for maintaining EDGE shown in the model suggest that the much more restricted mutational spectra observed in most natural adaptive mutation systems is itself adaptive. This evolutionary cost of mutagenesis readily explains the reduced mutational spectra observed in many natural systems, such as the peculiar molecular mechanism of tropism switching in Bordetella bacteriophage BPP-1 [39]. Mediated by reverse transcription, elevated mutagenesis is targeted to 23 adenosine bases (spanning 30 amino acid residues) at the C-terminal region of a protein that determines tropism. Both the adenosine-specific mutagenesis by reverse transcriptase and the precisely defined small window of mutagenesis target have to arise from strong specific selection.

And there are fitness consequences beyond those elucidated in the model. The potential for continued evolutionary innovation by EDGE is mainly contingent upon the selective conditions. EDGE could persist under conditions in which mutations in the EDGE target locus provide non-negligible net fitness benefits. Persistence is likely even if beneficial mutations occur at other loci. The locally high mutation rate of EDGE will generate beneficial mutations across multiple genetic backgrounds so that even selective sweeps driven by other loci are unlikely to purge EDGE. If EDGE net fitness benefits decline, however, EDGE persistence and innovation are in jeopardy. The EDGE genetic architecture itself (see Fig 1) is subject to mutation load, so that continuous purifying selection on the locus is necessary for its maintenance. This purifying selection is in addition to the necessity for beneficial mutations since the maintenance of the EDGE circuit does not necessarily provide direct fitness benefits. Stringent regulation of EDGE mutagenesis could potentially relax the need for purifying selection. For example, stressful conditions could turn on mutagenesis. Tight regulatory control would also reduce adaptive mutational benefits, because of lags in gene expression and phenotypic change associated with induction and turnover of gene products, respectively. Besides, mismatches in EDGE regulatory control and conditions in which EDGE mutagenesis is beneficial necessarily are the target of selection.

The EDGE system demonstrates the benefits of localized adaptive mutation for extended targeted evolution. EDGE provides insights on the benefits and costs of localized elevated mutation rates. Most adaptive microbial systems tightly constrain the variety of mutational variants, but this limitation is not a mechanistic requirement and may exist in part to reduce their fitness costs. Further development and use of EDGE will illuminate the complexity of evolving systems to adapt to natural selection. A limitation of the current work is the absence of a thorough mutational analysis of EDGE induced mutations. Such an analysis would further strengthen support for the system, and would be an important next step in its validation.

EDGE system differs from previous *in vivo* artificial mutagenesis systems in three important ways. First, EDGE localizes mutagenesis to the target region. Most other *in vivo* artificial systems affect large genomic regions, substantially reducing efforts in identifying the relevant mutation. Second, EDGE is not a repurposed natural system. There is no expectation of adaptive benefits beyond elevated mutation rates. Third, EDGE is a simple generic structure, in contrast with synthetic systems that involve extensive extracellular or experimentally interventionist steps [15]. While the construct design is novel, it uses common molecular components and processes from bacteria, in an arrangement similar to naturally occurring regulatory architectures (Fig 1). This simplicity simplifies the analysis, interpretation, and extends generality.

## Materials and methods

### Media and strains

Luria-Bertani medium (LB; 10 g Bacto tryptone, 5 g Bacto yeast extract, 10 g NaCl in 1 L ddH2O) with the addition of proper antibiotics were used to propagate cells. Fifteen grams per liter Bacto agar was added to prepare plates for isolating single colonies. Super Optimal Broth with catabolite repression medium (SOC; 20 g Bacto tryptone, 5 g Bacto yeast extract, 0.5 g NaCl, 0.186 g KCl, 0.952 g MgCl_2_, 1.204 g MgSO_4_, 3.603 g glucose in 1 liter) was used to recover cells from transformation. *E*. *coli* K-12 strain MM294 was used for plasmid construction, and strain MG1655 was used for testing mutagenesis and evolution experiments. Both strains were requested from Coli Genetic Stock Center, Yale.

### Plasmid construction

#### Accessory plasmid

The gene for Hin recombinase from bacterium *Salmonella enterica* was chemically synthesized through gBlock technology (Integrated DNA Technologies, Inc.). This fragment of DNA was PCR-amplified by a pair of primers. Phire Hot Start II DNA polymerase (Thermo Scientific, Inc.) was used for all PCR reactions in this study. Another pair of primers were used to amplify the backbone of plasmid pRD007 [40]. The products of PCR reactions were each purified by agarose gel extraction using GeneJET Gel Extraction Kit (Thermo Scientific, Inc.) following the manufacturer's protocol. The purified products were digested with restriction enzymes BamHI and NheI (FastDigest, Thermo Scientific, Inc.) following the manufacturer's protocol. The resulting digestion products were purified by agarose gel extraction before ligated using Rapid DNA Ligation Kit (Thermo Fisher Scientific, Inc.). The ligation product was purified using GeneJET Gel Extraction Kit (Thermo Scientific, Inc.) without running in agarose gel: the product was mixed with binding buffer, directly spun down a column, washed and eluted. The purified ligation product was then used to transform chemically competent MM294 cells following standard procedures in molecular biology [39]. After recovering in SOC medium, cells were plated on to LB agar plate supplemented with 50 μg/mL kanamycin (Sigma -Aldrich) and incubated at 37°C overnight. Colonies were checked by colony PCR using primers that target the gene for Hin recombinase. The colonies that gave a strong band with the correct size were used to inoculate a 5-mL LB culture. After 16 hours of growth at 37°C, shaken at 250 rpm, the plasmid was extracted using GeneJet Plasmid Plasmid Miniprep Kit (Thermo Fisher Scientific, Inc.). The plasmid product was eluted in water and saved at -20°C.

#### Target plasmid

Fusion PCR [41] was employed to assemble the multiple sequence components of the target genetic circuit into a single fragment of DNA. Several pairs of primers were used to PCR amplify *cI* from pKD46, *tetA-gfp* (a fusion gene between tetA and GFP) from a plasmid constructed previously, partial *sacB* from pRD007. About 30 bp overlap was designed between the 3' end of *the cI* product and the 5' end of the *tetA-gfp* product, and between the 3' end of *the tetA-gfp* product and the 5' end of the *sacB* product. So that, after agarose gel-purification, these three fragments were integrated with a final PCR reaction to yield a single long fragment. The backbone of petCOCO plasmid was also amplified and purified. Both fragments were digested with restriction enzymes NotI and NsiI (FastDigest, Thermo Scientific, Inc.), and agarose gel purified. The products were ligated and transformed into MM294, as described above. Here, LB agar plates were supplemented with 50 μg/mL ampicillin (Sigma-Aldrich, Inc.) to select for transformed cells. The candidate colonies were also treated as described above for confirmation and archive.

Site-directed mutagenesis was used to introduce the tetA D68N replacement [24] that inactivates tetracycline resistance. A pair of primers were designed to amplify the entire target plasmid; except one primer carries the single nucleotide substitution (G to A), and its 5' end was phosphorylated during chemical synthesis (Integrated DNA Technologies, Inc.). After agarose gel purification, the DNA was self-circularized using Rapid DNA Ligation Kit (Thermo Scientific, Inc.) and transformed into MM294 as described above. Here, LB agar plates were supplemented with 50 μg/mL ampicillin (Sigma-Aldrich, Inc.) to select for transformed cells. The candidate colonies were also treated as described above for confirmation and archive.

### Mutagenesis assay with apramycin or tetracycline

Since low doses of tetracycline can induce improved growth in *E*. *coli* [42], all selection experiments used a high dose of tetracycline (4 μg/mL). MG1655 cells were co-transformed with the target plasmid and the accessory plasmid. LB plates supplemented with 50 μg/mL ampicillin and 50 μg/mL Kanamycin were used to select double transformants. After 12–16 hours incubation at 37°C, single colonies were transferred using pipette tips each into 50 μl LB medium and re-suspended. Two to five μl of this suspension was inoculated into a glass tube (13×100 mm; Fisher Scientific) with one mL LB supplemented with 50 μg/mL ampicillin and 50 μg/mL Kanamycin, with or without 150 μM IPTG. The glass tube was loosely capped for aeration while preventing contamination. The inoculated cultures were incubated at 37°C, shaken at 250 rpm, for 12 hours to full density. Fifty μl from each culture, corresponding to 10^8^ cells, were plated onto LB plate supplemented with 4 μg/mL tetracycline. Each culture was diluted one thousand-fold, and 50 μl was plated, corresponding to 10^5^ cells, onto LB plate supplemented with 4 μg/mL apramycin. The plates were incubated at 37°C 12–16 hours for colonies to emerge. The number of colonies each plate was counted. Four μg/mL was substantially higher than the MIC of tigecycline, which was below 0.1 micrograms/microliter, and thus lethal to *E*. *coli* cells. During selection, this lethal concentration was applied to avoid physiological and/or genetic complications associated with a sub-lethal concentration of antibiotics. A selection of colonies was each inoculated to a 50 mL LB culture supplemented with 50 μg/mL ampicillin for plasmid extraction using ZymoPure Plasmid Midiprep Kit (Zymo Research). The plasmids were each transform into a fresh batch of MG1655 competent cells and plated on an LB plate supplemented with 4 μg/mL tetracycline to check if the tetracycline resistance was plasmid-borne.

### Statistical analysis of induced mutation

We used a fixed-effects two-way ANOVA to assess the effects of EDGE induction on mutation rate to tetracycline (gain-of-function), apramycin (loss-of-function) and streptomycin (off-target). Colony counts were transformed by log_10_(count + 10) to normalize variances across treatments [43]. Main effects were Trait (gain, loss, off-target) and Induction (on, off) with Trait-by-Induction as an interaction term. The ANOVA had an adjusted R^2^ of 91.3%, indicating that it captured most of the variance in the data. Analysis of the three induction experiments was carried out by individual planned comparisons for each, which was supported by an overall partial F-test (*F*_3,24_ = 48.13, p = 2.64 x 10^−10^).We used a bootstrap analysis to specifically compare the effects of EDGE induction on gain of tetracycline versus streptomycin resistance [44]. We bootstrapped the fold-increase of mutations due to EDGE induction for tetracycline and streptomycin resistance. We calculated the 95% confidence interval by estimating the 2.5% and 97.5% limits of difference in fold-increase (see S3 Fig for a description).

### Flow cytometer check of GFP expression

FACSCalibur (BD Sciences) was used to analyze cells for GFP expression. Cells acquired directly from colonies or cultures were diluted in flow buffer (7 g K_2_HPO_4_, 2 g KH_2_PO_4_ in 1 liter). An air-cooled argon-ion laser with wavelength 488 nm was used to excite GFP. The following parameter values were used for optimal data acquisition for bacteria and the low-level GFP expression typical of cells in this study: FSC, 01; SSC: 396; FL1, 750. For each sample, 50 000 events were collected. The raw FCS files were analyzed with software FlowJo^®^.

### Evolution of tetA for resistance to tigecycline

MG1655 cells were prepared as per the mutagenesis assay. After 12 hours of growth and induction in LB supplemented with 150 μM IPTG, 100 μl were plated onto a series of LB plates with graded concentrations of tigecycline (0.8, 1.2, 1.6, 2.0, 3.0 μg/mL). The plates were incubated at 37°C for 16–20 hours. This experiment was done in triplicate. The colonies that emerged were checked with a flow cytometer for GFP signal. Five colonies were sub-cultured in liquid, and target plasmids were extracted. The target plasmids were individually transformed into wildtype *E*. *coli* to verify that resistance to tigecycline was carried on target plasmid rather arising from the chromosome. None of the five were false positive (resistance arising from the chromosome). The tetracycline fragment on the target plasmid was amplified by PCR, and the resulting DNA was Sanger sequenced (University of Minnesota Genomics Center). This sequencing confirmed that the tetracycline resistance gene had been repaired.

### Model of fitness effects of different mutators

#### Differing number of allelic states

We model the competition of two mutational systems, one of which has the optimal number alleles (n), and another with an excess number of alleles (x, x > n). The environment fluctuates among n states, with lethal selection for individuals having the “wrong” matching allele. This is a haploid model. In this system, there are no fitness consequences associated with the mutational systems besides the lethal selection (i.e., no growth rate differences). Lethal selection occurs only when the population is at carrying capacity; otherwise, the population is growing. Mutations occur in every locus in the population, albeit 1/n and 1/x, respectively “mutate” to the same allelic state as they had originally (see S4 Fig for a graphical description).

In populations experiencing death, the relative fitness of genotypes is best modeled by the Selection Rate Constant (r), which is the difference in malthusian parameters [30, 31]. For one bout of selection in our model, this is

$$r_{N/X}=ln(\frac{N_{1}}{N_{0}})-ln(\frac{X_{1}}{X_{0}}),$$ (1)

where N and X are the population sizes of the mutators with either n or x mutant allelic states, following a bout of mutation (0) and after regrowth to carrying capacity (1). If f and (1-f) are the initial frequencies of the mutators with n and x alleles prior to mutation, respectively. The population size of the mutators with n alleles after one bout of selection is given by

$$N_{1}=N_{0}2^{d}=\frac{f}{n}2^{d},$$ (2)

“d” is the number of divisions required for the entire population (all genotypes) to recover to carrying capacity. The growth rate of all genotypes is identical, (selection is only against genotypes having the wrong matching allele), the number of divisions following selection is the same for all surviving mutators. Thus,

$$r_{N/X}=ln(\frac{f\frac{1}{n}2^{d}}{f})-ln(\frac{(1-f)\frac{1}{x}2^{d}}{1-f})=ln(\frac{x}{n}).$$ (3)

The ratio (x/n) is the ratio of possible alleles, excess over optimal.

#### Differing mutation rates for the optimal number of alleles

For a mutator locus with the optimal number of potential alleles, a sufficiently high mutation rate is necessary to generate mutations. The optimal mutation rate is that which generates the largest number of matching allelic variants (1/n), which is the same as for each allelic variant. We compare two mutational systems (A and B), both having the optimal number of alleles (n). However, the mutation rates differ among the loci, *μ*_*A*_ and *μ*_*B*_, respectively (0 < *μ*_*B*_ < *μ*_*A*_ ≤ 1). Following from above,

$$r_{A/B}=ln(\frac{N_{A_{1}}}{N_{A_{0}}})-ln(\frac{N_{B_{1}}}{N_{B_{0}}}),$$ (4)

$$r_{A/B}=ln(\frac{f\frac{\mu_{A}}{n}2^{d}}{f})-ln(\frac{(1-f)\frac{\mu_{B}}{n}2^{d}}{1-f})=ln(\frac{\mu_{A}}{\mu_{B}}).$$

Simultaneously considering both the number of potential alleles and differing mutation rates

$$r_{N_{A}/X_{B}}=ln(\frac{f\frac{\mu_{A}}{n}2^{d}}{f})-ln(\frac{(1-f)\frac{\mu_{B}}{x}2^{d}}{1-f})=ln(\frac{x_{B}}{n_{A}}\frac{\mu_{A}}{\mu_{B}}).$$ (5)

## Supporting information

S1 Fig Mutation rates of selected Hin endonuclease activity. Selected variants produce a greater number of mutant colonies, from 30 to 270% more.
(PDF)

S2 Fig Solved structure for a homologue to Hin recombinase. Location of A76P substitution in Hin variant with increased mutation rate. The ribbon diagram shows the structure of a homolog of Hin recombinase: a homodimer of γδ resolvase in complex with DNA (pdb id: 1gdt). Cleavage of the substrate DNA occurs when two homodimers, each in complex with its substrate DNA, join to form a tetramer (Yang & Steitz 1995). The homologous residue to the alanine 76 of Hin recombinase is highlighted by black. The substitution of this residue by proline (Mutant 5) likely disrupts the α helix required for the formation of a tetramer. Chang & Johnson (2015) suggested that disruption of this region may disrupt inhibition of DNA cleavage by the homodimer. Since the formation of the tetramer is the rate-limiting step of Hin recombinase, the elimination of that requirement is expected to increase the rate of cleavage.[45, 46].
(PDF)

S3 Fig The effect of EDGE induction was calculated for the difference between tetracycline and streptomycin. The fold-increase in mutation rate due to EDGE induction was assessed by bootstrapping 10,000 times, for both tetracycline and streptomycin. Bootstrapping was done in a pairwise manner, so that a 95%CI could be determined based on the distribution of the difference (tetracycline—streptomycin).
(PDF)

S4 Fig Mutational systems with n (left) or x (right) possible alleles and only n environments (x > n). Shaded areas are beneficial allele—environment combinations. For any one environment, there is only one non-zero fitness allele. Hence the population is reduced to 1/n or 1/x of the initial population size at every bout of selection.
(PDF)

S1 Table Hin recombinase genotypes obtained by selection for increased mutation rate. (PDF)
