# Supplementary figures and images for: Evolutionary innovation using EDGE, a system for localized elevated mutagenesis
Source: PLoS One. 2020 Apr 30;15(4):e0232330. doi: 10.1371/journal.pone.0232330 (PMC7192385; doi:10.1371/journal.pone.0232330)

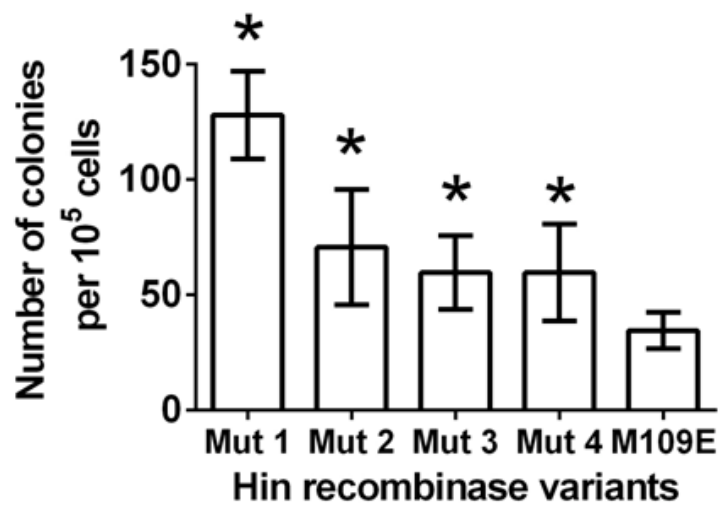

Supplement: S1 Fig — Selected variants produce a greater number of mutant colonies, from 30 to 270% more. (PDF) [file pone.0232330.s001.pdf]

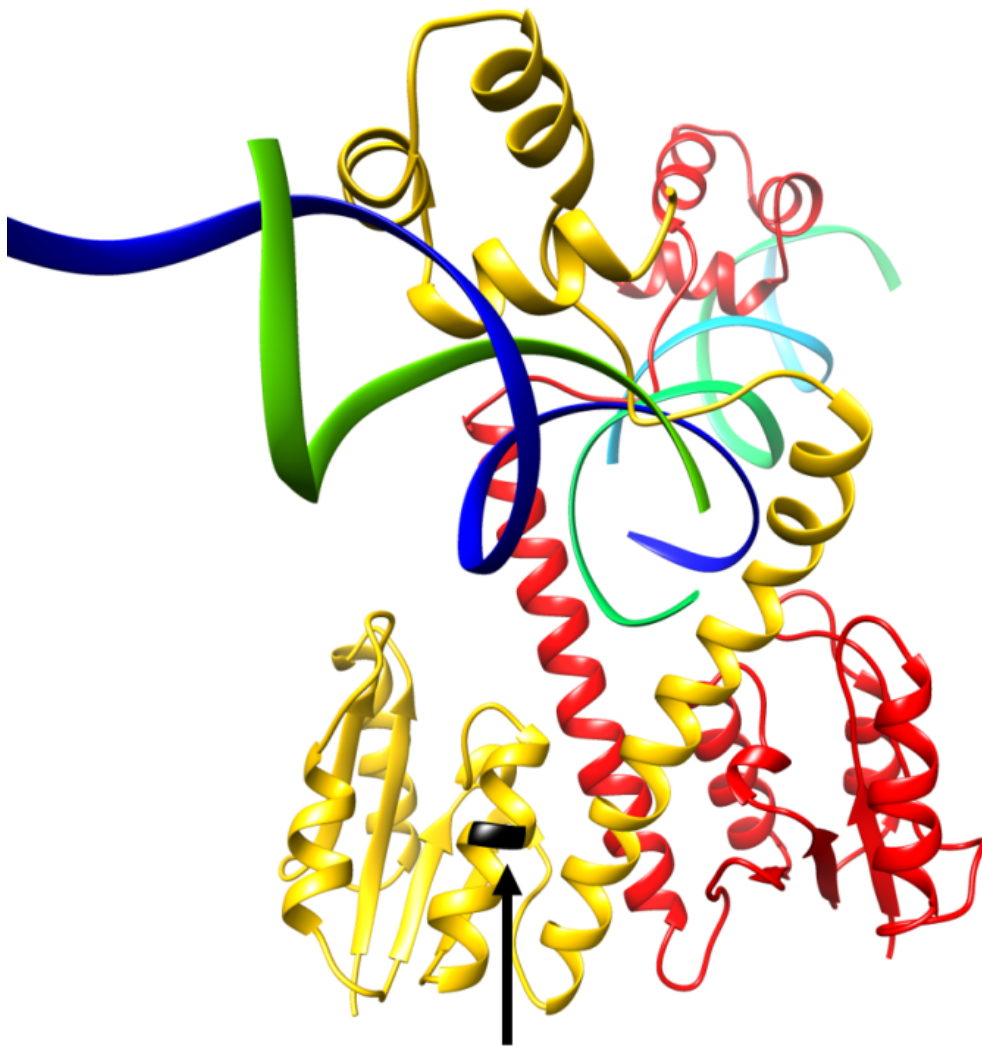

---

**Substitution of alanine with proline**

Supplement: S2 Fig — Location of A76P substitution in Hin variant with increased mutation rate. The ribbon diagram shows the structure of a homolog of Hin recombinase: a homodimer of γδ resolvase in complex with DNA (pdb id: 1gdt). Cleavage of the substrate DNA occurs when two homodimers, each in complex with its substrate DNA, join to form a tetramer (Yang & Steitz 1995). The homologous residue to the alanine 76 of Hin recombinase is highlighted by black. The substitution of this residue by proline (Mutant 5) likely disrupts the α helix required for the formation of a tetramer. Chang & Johnson (2015) suggested that disruption of this region may disrupt inhibition of DNA cleavage by the homodimer. Since the formation of the tetramer is the rate-limiting step of Hin recombinase, the elimination of that requirement is expected to increase the rate of cleavage.[45, 46]. (PDF) [file pone.0232330.s002.pdf]

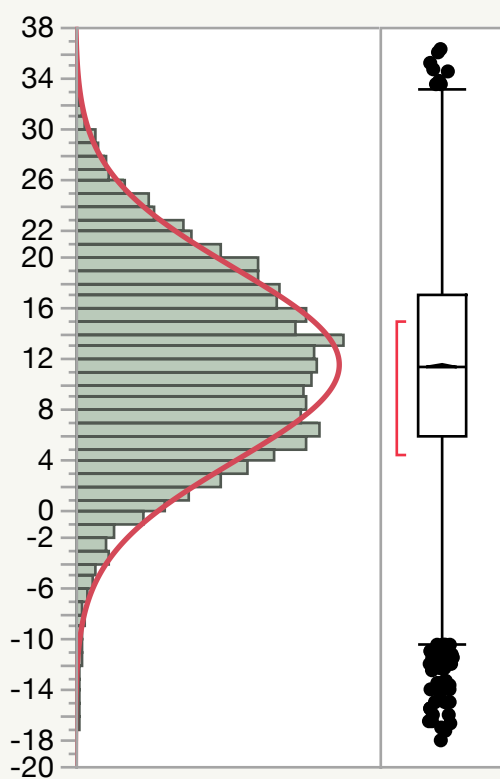

|        |          |             |
|--------|----------|-------------|
| 100.0% | maximum  | 36.25       |
| 99.5%  |          | 29.75       |
| 97.5%  |          | 25.83333333 |
| 90.0%  |          | 21.33333333 |
| 75.0%  | quartile | 16.96875    |
| 50.0%  | median   | 11.5        |
| 25.0%  | quartile | 6           |
| 10.0%  |          | 2           |
| 2.5%   |          | -3          |
| 0.5%   |          | -10         |
| 0.0%   | minimum  | -18         |

N

10000

Supplement: S3 Fig — The fold-increase in mutation rate due to EDGE induction was assessed by bootstrapping 10,000 times, for both tetracycline and streptomycin. Bootstrapping was done in a pairwise manner, so that a 95%CI could be determined based on the distribution of the difference (tetracycline—streptomycin). (PDF) [file pone.0232330.s003.pdf]

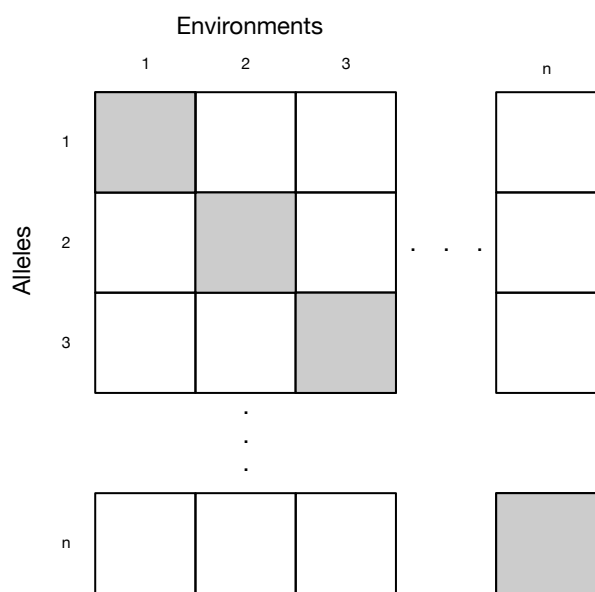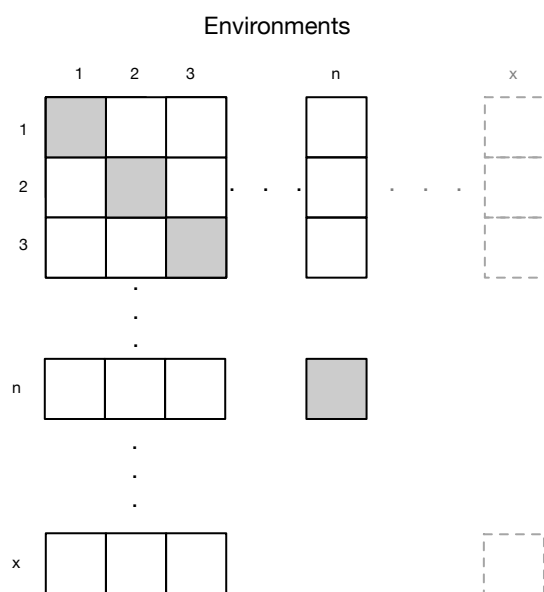

Supplement: S4 Fig — Shaded areas are beneficial allele—environment combinations. For any one environment, there is only one non-zero fitness allele. Hence the population is reduced to 1/n or 1/x of the initial population size at every bout of selection. (PDF) [file pone.0232330.s004.pdf]
