# Supplementary material for: Evolutionary innovation using EDGE, a system for localized elevated mutagenesis
Source: PLoS One. 2020 Apr 30;15(4):e0232330. doi: 10.1371/journal.pone.0232330 (PMC7192385; doi:10.1371/journal.pone.0232330)
Supplement: S1 Table — (PDF) [file pone.0232330.s005.pdf]

Table S1. Hin recombinase genotypes obtained by selection for increased mutation rate

**Mutant Index**	**Mutations**
1	wildtype
2	R8G
3	K39M
4	A143V, L176I
5	A76P
